# Supplementary material for: Effects of Climatic Factors on Diarrheal Diseases among Children below 5 Years of Age at National and Subnational Levels in Nepal: An Ecological Study
Source: Int J Environ Res Public Health. 2022 May 18;19(10):6138. doi: 10.3390/ijerph19106138 (PMC9140521; doi:10.3390/ijerph19106138)
Supplement: Supplementary file 1 [file ijerph-19-06138-s001.zip › ijerph-1678279-supplementary.pdf]

Supplementary Materials

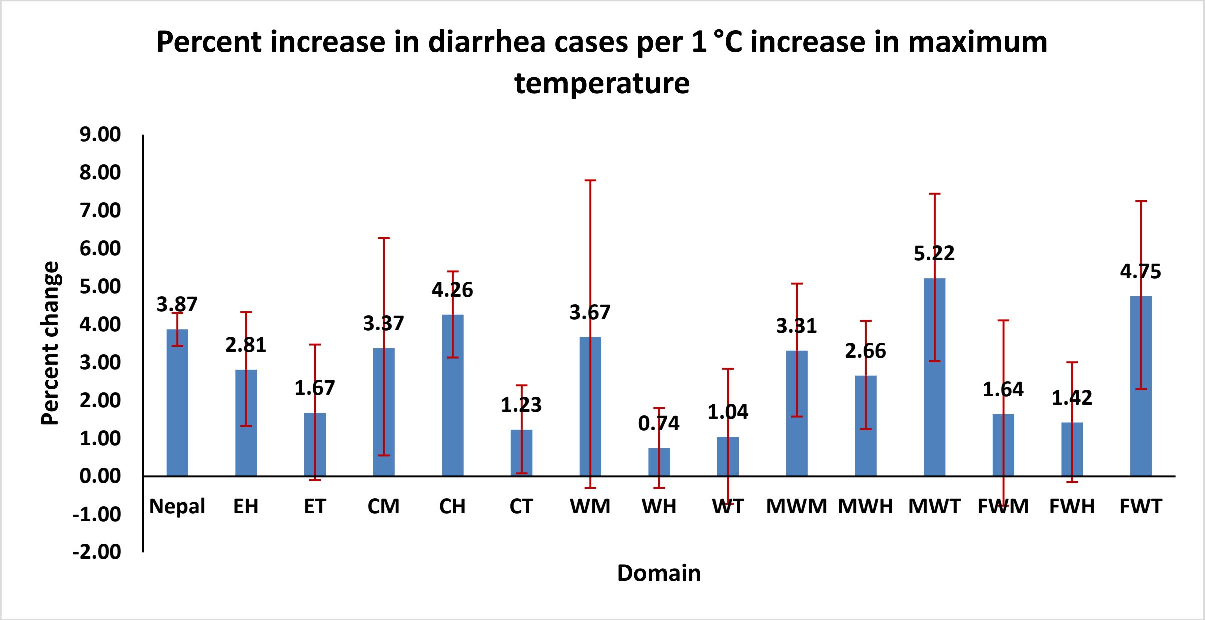

Supplementary Figure S1. Percentage increase in diarrheal cases per 1 °C increase in maximum temperature.

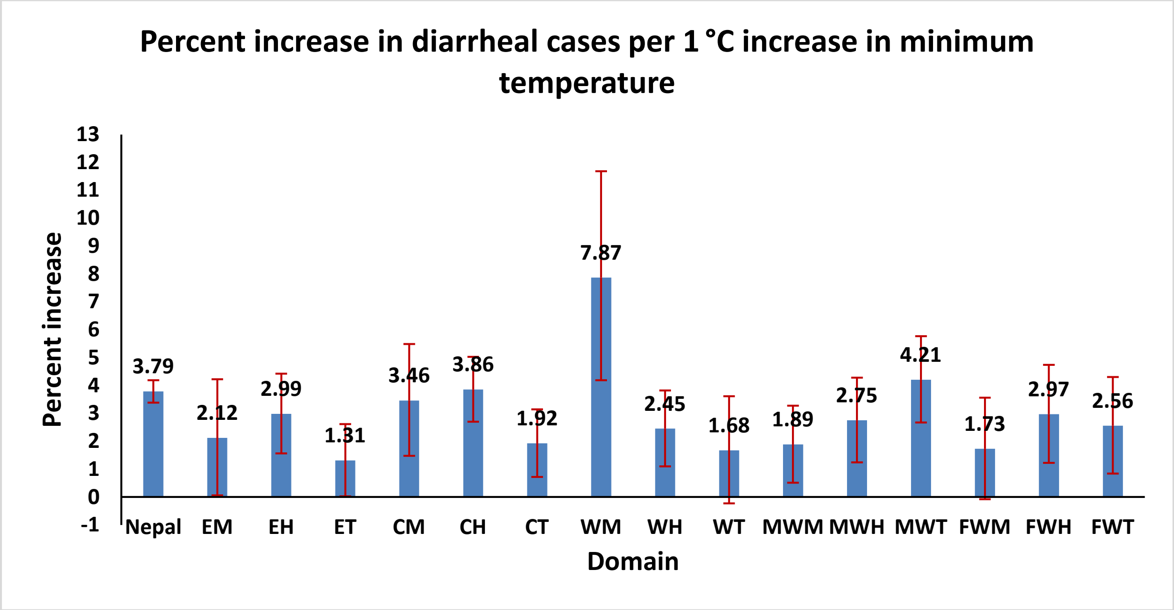

Supplementary Figure S2. Percentage increase in diarrheal cases per 1 °C increase in minimum temperature.

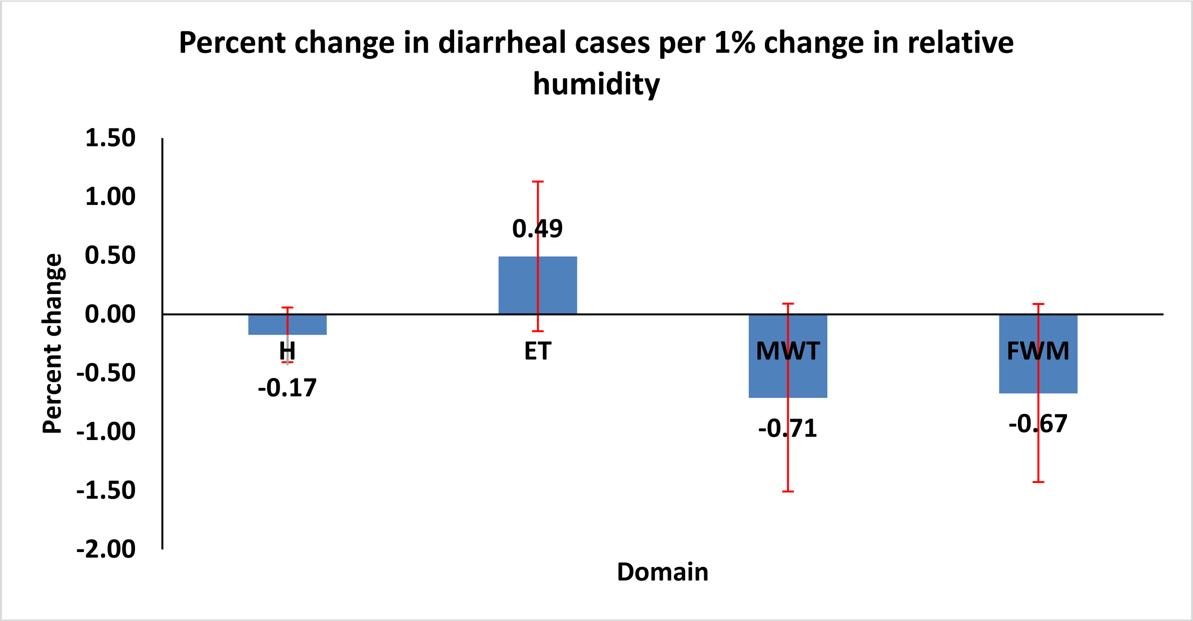

Supplementary Figure S3. Percentage change in diarrheal cases per 1% increase in relative humidity.

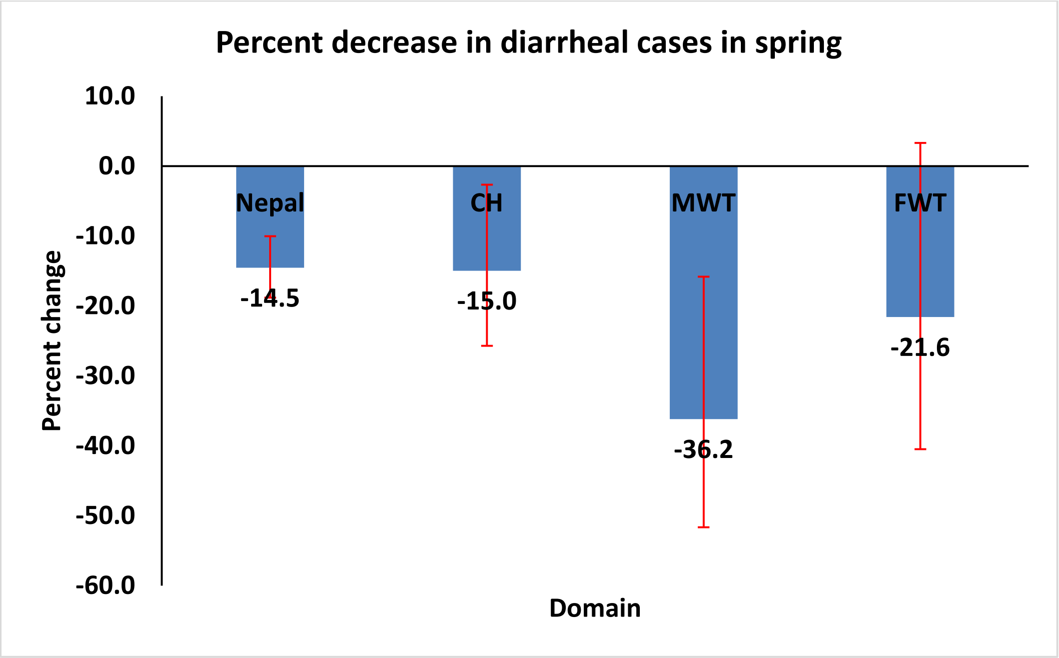

Supplementary Figure S4. Percentage decrease in diarrheal cases in spring season with reference to summer.

Note: Data in the chart only include the domains that show significant effect.

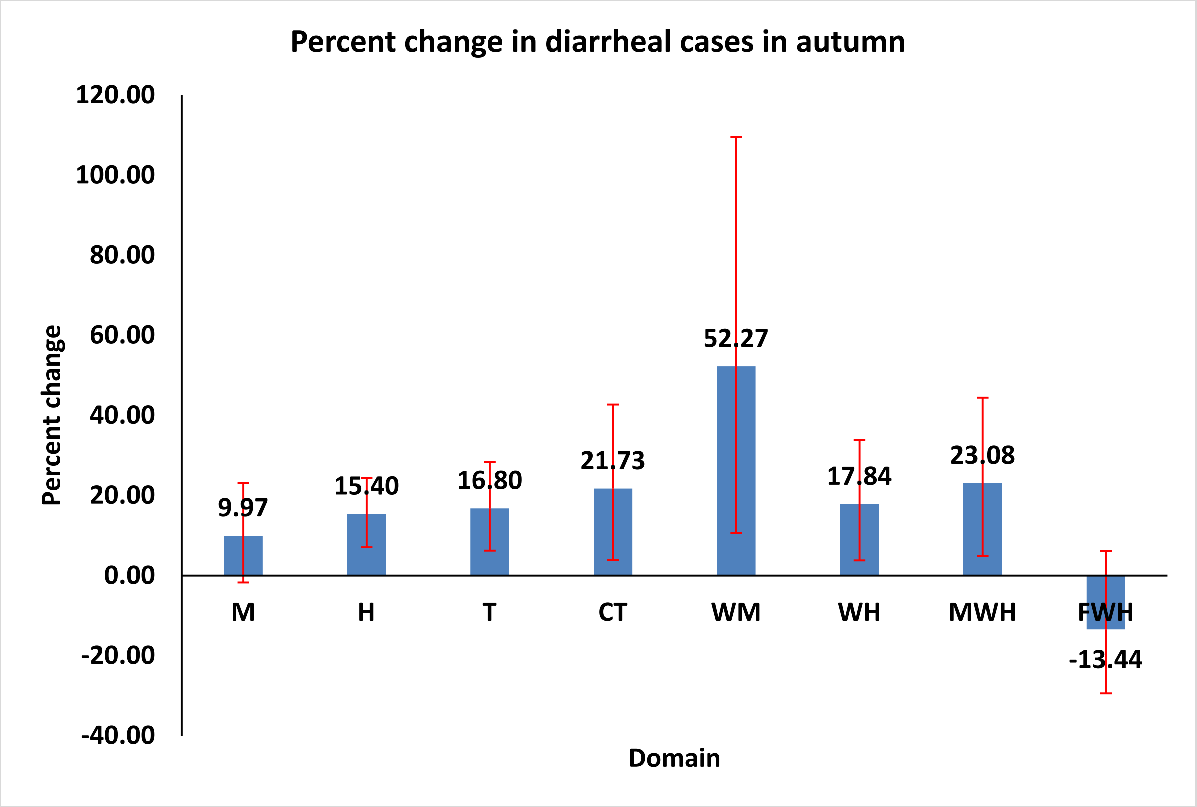

Supplementary Figure S5. Percentage change in diarrheal cases in autumn season with reference to winter.

Note: Data in the chart only include the domains that show significant effect.
